# A novel technique using chronic infusion of small extracellular vesicles from gestational diabetes mellitus causes glucose intolerance in pregnant mice

**DOI:** 10.1042/CS20220484

**Published:** 2022-11-03

**Authors:** Laura B James-Allan, Frederick J Rosario, Lana Madi, Kelsey Barner, Soumyalekshmi Nair, Andrew Lai, Flavio Carrion, Theresa L Powell, Carlos Salomon, Thomas Jansson

**Affiliations:** 1 Division of Reproductive Sciences, Department of Obstetrics/Gynecology, University of Colorado, Aurora, CO, U.S.A.; 2 Exosome Biology Laboratory, Centre for Clinical Diagnostics, UQ Centre for Clinical Research, Royal Brisbane and Women’s Hospital, Faculty of Medicine and Biomedical Sciences, The University of Queensland, Australia; 3 Departamento de Investigación, Postgrado y Educación Continua (DIPEC), Facultad de Ciencias de la Salud, Universidad del Alba, Santiago, Chile; 4 Department of Pediatrics, University of Colorado, Aurora, CO, U.S.A.

**Keywords:** glucose homeostasis, insulin resistance, microparticles, pancreatic beta cell

## Abstract

Small extracellular vesicles (sEVs) play a central role in cell-to-cell communication in normal physiology and in disease, including gestational diabetes mellitus (GDM). The goal of the present study was to test the hypothesis that chronic administration of sEVs isolated from GDM causes glucose intolerance in healthy pregnant mice. Small EVs were isolated from plasma between 24 and 28 weeks gestation from healthy pregnant women (controls) and GDM, and infused intravenously for 4 days in late pregnant mice using a mini-osmotic pump. Subsequently *in vivo* glucose tolerance was assessed, and muscle and adipose tissue insulin sensitivity and islet glucose stimulated insulin secretion (GSIS) were determined *in vitro*. Mice infused with sEVs from GDM developed glucose intolerance. Administration of sEVs from controls, but not sEVs from GDM women, stimulated islet GSIS and increased fasting insulin levels in pregnant mice. Neither infusion of sEVs from controls nor from GDM women affected muscle insulin sensitivity, placental insulin or mTOR signaling, placental and fetal weight. Moreover, these results were not associated with immunomodulatory effects as human sEVs did not activate mouse T cells *in vitro*. We suggest that circulating sEVs regulate maternal glucose homeostasis in pregnancy and may contribute to the attenuated islet insulin secretion and more pronounced glucose intolerance in GDM as compared with healthy pregnancy.

## Introduction

Gestational diabetes mellitus (GDM) is defined as glucose intolerance with onset during pregnancy [1]. The prevalence of GDM worldwide is increasing with recent reports of 2–10% of pregnant women developing GDM [2]. GDM is associated with fetal overgrowth, neonatal hypoglycaemia, and elevated perinatal morbidity and mortality. Women with gestational diabetes are more likely to develop GDM in future pregnancies, and both women and their offspring have a higher risk of cardiovascular and metabolic disease later in life [3–6].

Extracellular vesicles (EVs) are membrane bound particles secreted from many cell types that contain bioactive molecules including proteins, messenger (mRNA) and microRNA (miRNA). Small EVs (sEVs) ranging from ∼50 to 150 nm in size include exosomes [7], produced from the late endosomal pathway and released into the extracellular compartment upon fusion of multivesicular bodies (MVB) with the plasma membrane, and microvesicles, formed from outward budding and fission with the plasma membrane [8–10]. sEVs are believed to play a central role in cell-to-cell communication in normal physiology and in disease [11–15], including in pregnancy [16,17]. Placental sEVs can be isolated from maternal blood using placental alkaline phosphatase (PLAP) as a marker [16–20]. We have demonstrated that placental sEVs can be detected in the maternal circulation as early as 6 weeks of gestation [21,22] and that placental sEVs constitute ∼20% of total (all) sEVs in the maternal circulation at term [21]. Recently, we reported that the levels of circulating sEVs (total and placenta-derived) are higher in GDM compared with normal pregnancies across gestation [23].

Normal pregnancy is critically dependent on the development of maternal insulin resistance and an increased capacity to secrete insulin, which allows for the allocation of nutrients for fetal growth [24,25]. In pregnancies complicated by GDM, maternal insulin resistance is increased further and β-cell compensation is inadequate, resulting in various degrees of fasting hyperglycemia [26]. A number of diverse mechanisms, including impaired secretion of placental hormones [27–29], β-cell dysfunction caused by chronic fuel excess [30] and β-cell toxicity [31], defects in the skeletal muscle insulin signaling pathway [32,33] as a result of elevated levels of saturated fatty acids [34] and proinflammatory cytokines [35] and changes in the gut microbiome [36] have been proposed as mechanisms underpinning GDM. However, conclusive evidence to support a critical role for these mechanisms in the development of GDM in women are lacking. Interestingly, EVs offer new insights into treating endocrine disorders such as GDM [37].

We have previously shown that human placental sEVs isolated from women with GDM at term carry a specific set of miRNAs associated with skeletal muscle insulin signaling [38]. In early gestation there is differential expression of miRNAs in EVs between GDM and normal pregnancies, including miRNAs involved in insulin secretion/regulation and glucose transport [39]. These findings suggest that the increased concentration of sEVs observed in GDM women may be linked to aberrant maternal insulin and glucose tolerance.

We previously reported that sEVs in maternal circulation during pregnancy contribute to the metabolic adaptations that occur in normal pregnancy and glucose intolerance observed in GDM [40]. However, the effect of sEVs from healthy pregnant women and women with GDM on pregnant mice is unknown. We hypothesized that chronic administration of sEVs isolated from women with GDM causes glucose intolerance in normal pregnant mice. To address this hypothesis, sEVs isolated from plasma of healthy pregnant women and women with GDM, collected at the time of diagnosis at 24–28 weeks of gestation, were infused intravenously for 4 days in late pregnant mice using a mini-osmotic pump. At 17.5 days of gestation, *in vivo* glucose tolerance was assessed, muscle and adipose tissue insulin sensitivity and islet glucose stimulated insulin secretion (GSIS) was determined *in vitro*. The activity of placental insulin and mTOR signaling, and placental and fetal weights, were determined. Additionally, the immunomodulatory effects of sEVs were assessed by T-cell activation and proliferation *in vitro*.

## Methods

### Participants and sample collection

The study was approved by the Human Research Ethics Committees of the Royal Brisbane and Women’s Hospital and the University of Queensland (HREC/11/QRBW/342). Written informed consent was obtained from healthy non-pregnant and pregnant women 18–35 years of age, representing the ethnic mix of the overall population in Brisbane (∼86% Non-Hispanic White, 8% Asian and 6% Middle Eastern). All pregnant women were screened for GDM and were diagnosed according to the then current criteria of the Australasian Diabetes in Pregnancy Society (ADIPS): fasting venous plasma glucose concentration of ≥5.5 mmol/l glucose and/or ≥8.0 mmol/l glucose 2 h after a 75 g oral glucose load at 24–28 weeks gestation. Fasting antecubital venous blood samples were collected in EDTA tubes at the time of the diagnostic OGTT. Smoking and substance abuse were exclusion criteria for all women. Exclusion criteria for pregnant women included in the isolation of sEVs were twin gestation, premature rupture of membranes, placental abruption, fetal congenital and genetic abnormalities, pre-gestational diabetes, thyroid disorder, chronic hypertension, preeclampsia and chorioamnionitis. Samples used were matched for age, weight, body mass index and gestational age. Selected clinical characteristics of the study subjects and their infants have been previously published [40].

### Isolation of sEVs

sEVs were isolated from 1 ml plasma as previously described [40]. In brief, sEVs were isolated by differential centrifugation and the pellet resuspended and layered on the top of a discontinuous iodixanol gradient. Fractions were collected manually from top to bottom, diluted with PBS and centrifuged at 100,000 ***g***for 2h at 4°C. Finally, the pellet containing the enriched sEV population was resuspended in PBS. sEVs were characterized, according to the recommendation of the International Society of Extracellular Vesicles [41], by size distribution (Nanosight N500), morphology and abundance of proteins associated with sEVs. We have previously established that sEVs are stable when stored at −80°C [22]. Completely de-identified sEVs were shipped to the University of Colorado on dry ice for the *in vivo* experiments.

### Characterization of sEVs

#### Nanoparticle tracking analysis

Nanoparticle tracking analysis (NTA) measurements were performed using a NanoSight NS500 instrument (NanoSight NTA 2.3 Nanoparticle Tracking and Analysis Release Version Build 0033) following the manufacturer’s instructions and as previously described [23]. In brief, the NanoSight NS500 instrument measures the rate of Brownian motion of nanoparticles and consists of a light scattering system that provides a reproducible platform for specific and general nanoparticle characterization (NanoSight Ltd., Amesbury, U.K.). Samples were processed in duplicate and diluted with PBS over a range of concentrations to obtain 10–100 particles per image (optimal ∼50 particles per image) before analysis. Samples were added into the chamber (temperature: 25°C and viscosity: 0.89 cP) and the camera level set to obtain an image that has sufficient contrast to clearly identify particles while minimizing background noise (camera level: 10 and capture duration: 60 s). The capture videos (two videos per sample) were processed and analysed. A combination of high shutter speed (600) and gain (250) followed by manual focusing enables optimum visualization of a maximum number of vesicles. We included a minimum of 200 tracks completed per video in duplicate. An Excel spreadsheet (Microsoft Corp., Redmond, Washington) was also automatically generated, showing the concentration at each particle size.

#### Validation of sEVs by immunoblotting

sEV proteins were separated by polyacrylamide gel electrophoresis and subsequently transferred to Immobilon-®FL polyvinylidene difluoride membranes (Millipore, Billerica, MA, U.S.A.) and probed with CD63 (sc15363, Santa Cruz Biotechnology, TX, U.S.A.), CD9 (sc13118, Santa Cruz Biotechnology) and TSG101 (EPR7130, Abcam, Cambridge, U.K.) and absence of a negative control Grp94 (20292T, Cell Signaling Technology, MA, U.S.A.).

#### Electron microscopy

sEVs isolated by differential and buoyant density gradient centrifugation were assessed by transmission electron microscopy. sEVs pellets (as described above) were fixed in 3% (w/v) glutaraldehyde and 2% paraformaldehyde in cacodylate buffer, pH 7.3. Five microliters of sample was then applied to a continuous carbon grid and negatively stained with 2% uranyl acetate. The samples were examined in an FEI Tecnai 12 transmission electron microscope (FEI™, Hillsboro, Oregon, U.S.A.).

#### Single particle analysis

Particle distribution and concentration were obtained using ExoView R100 (Nanoview Biosciences) analysis as previously described [42]. Briefly, binding of EVs to the microarray chip (EV-TETRA-P) coated with different antibodies, including anti-CD9, anti-CD81, and anti-CD63, ensures measured particles express specific surface components. ExoView has a high sensitivity and can accurately measure single EV particles ∼50 nm diameter using single-particle interferometric reflectance imaging. About 2.5 μg EVs were diluted in 500 μl incubation solution and 40 μl placed on a chip to incubate overnight. Following three washes with incubation solution, the chips were then incubated with antibodies (CD9, CD81, and PLAP, 1:1200 dilution) at room temperature for 1 h followed by one wash in incubation solution and three washes with wash solution. Chips were rinsed and then analyzed (ExoView R100). Chips coated with each antibody were prepared in triplicate and the whole chip was scanned for particle analysis. Detection of Placental Alkaline Phosphatase (PLAP) on circulating plasma-derived sEVs was performed using anti-PLAP, labeled using Alexa Fluor 555 Protein Labeling Kit (A20174, Molecular Probes) according to the manufacturer’s instructions.

### *In vivo* mouse model

All protocols were approved by the Institutional Animal Care and Use Committee at the University of Colorado Anschutz Medical Campus (#00112). Animals were anaesthetised using isoflurane. Adult mice were euthanised by CO_2_ followed by cervical dislocation and fetuses were killed by decapitation. Pregnant female C57BL/6 mice (*n*=32) were anesthetized on day 13.5 of pregnancy, and the right jugular vein was cannulated using a sterile polyurethane catheter (Alzet, 0007700) and secured in place with sutures. An Alzet mini-osmotic pump (model 1003D, 100 µl, infusion rate 1 µl/h), filled with either PBS or human sEVs was connected to the jugular vein catheter and placed subcutaneously in the scapular region using a small incision. Total sEVs were isolated from individual women (healthy pregnancy, *n*=24 and GDM, *n*=24) and subsequently pooled according to the treatment groups. EVs were quantified by Nanoparticle Tracking Analysis (NTA) using The NanoSight instrument as previously described [23,40]. To assess the reproducibility of the quantification method, synthetic nanoparticles, and an EV pool with a % CV range of 4.2–7.5 were used. The final concentration of sEVs in the pooled samples was measured and the same number of sEVs (2.7 × 10^12^ sEVs) from each group was resuspended in 100 µl PBS and loaded into osmotic pumps and infused continuously over 4 days. PBS without sEVs was used as a control. Mice were housed one per cage under controlled conditions (25°C, 14:10 h light–dark cycle). On day 4, mice were fasted for 1 h and a glucose tolerance test (GTT) was carried out. After a fasted baseline blood glucose level was determined by tail-vein blood, blood glucose was measured repeatedly (15, 30, 60 and 90 min) after an intraperitoneal injection of glucose (2 g/kg). Mice were fasted for an additional hour after the last blood glucose measurement. Mice were killed, and arterial blood was collected in EDTA tubes by cardiac puncture. The pancreas, adipose tissue and skeletal muscle were collected from the dams. Fetuses and corresponding placentas were collected and weighed.

### Pancreatic islet isolation and measurement of glucose stimulated insulin secretion

The pancreas was digested via ductal perfusion with 10 mg/ml Collagenase P (Roche, Switzerland) in Hanks balanced salt solution supplemented with 4 mM NaCO_3_ and 1% (v/v) bovine serum albumin (BSA). Subsequently, the pancreas was excised and digested for 15 min at 37°C. Tissue was then washed in cold supplemented Hanks balanced salt solution without collagenase. Islets were isolated by histopaque gradient centrifugation and washed. Isolated islets (50/sample) were cultured in RPMI medium for 2 h, pre-conditioned in Krebs–Ringer bicarbonate buffer for 90 min, and then incubated for 60 min with either 2.8 or 16.7mM glucose in the same buffer. After 60 min, the supernatant was collected and stored at −80°C for later analysis of insulin content by ELISA (Alpco, NH, U.S.A.).

### Measurements of plasma insulin and adiponectin

The concentration of insulin and adiponectin in mouse plasma was determined using colorimetric ELISAs (Alpco), following instructions provided by the manufacturer.

### Insulin stimulated IRS-1 and Akt phosphorylation in skeletal muscle

Gastrocnemius muscle strips were dissected, washed and preincubated for 30 min with Krebs-Henseleit bicarbonate buffer (120 mM NaCl, 4.7 mM KCl, 1.25 mM MgSO_4_, 1.2 mM KH_2_PO_4_, 2.5 mM CaCl_2_ and 25 mM NaHCO_3_, pH 7.4) containing 5.5 mM glucose, 2 mM sodium pyruvate, and 0.1% (v/v) BSA followed by incubation with and without insulin (10 mU/ml, Sigma-Aldrich, St Louis, MO) for 5 min. The media were gassed continuously with 95% O_2_ and 5% CO_2_. After 5 min of incubation, muscle strips were blotted rapidly on filter paper, frozen in liquid nitrogen and stored at −80°C until analysis. Total protein expression and phosphorylation of key components in the insulin signaling pathway were measured by Western blot.

### Assessment of insulin signaling activity

Briefly, skeletal muscle, adipose and placental tissues were homogenized in Buffer D containing protease and phosphatase inhibitors and protein (20 µg) were loaded on to precast polyacrylamide gels (Bio-Rad, CA, U.S.A.). After electrophoresis, the proteins were transferred to PVDF membranes overnight at 4°C. Following transfer, membranes were blocked in either 5% (w/v) milk in Tris-buffered saline with 0.1% (v/v) Tween80 (TBS-T) or 5% (w/v) BSA in TBS-T. Membranes were incubated with primary antibodies: IRβ (SC-135949, Santa Cruz Biotechnologies), Akt (4691, Cell Signaling Technology), Akt-Thr308 (05-802 R, MilliporeSigma), Akt-Ser473 (9271, Cell Signaling Technology), S6rp (2217, Cell Signaling Technology), pS6rp-Ser235/236 (2211, Cell Signaling Technology), 4EBP1 (9452, Cell Signaling Technology) and p4EBP1-Ser65 (9451, Cell Signaling Technology). Membranes were incubated with appropriate peroxidase labeled secondary antibody (Cell Signaling Technology). Bands were visualized using enhanced chemiluminescence detection reagents (Thermo Fisher Scientific, MA, U.S.A.). Densitometry analysis was performed using GeneTools (4.3.8 Syngene, Cambridge, U.K.). Amido black total protein staining of the membrane was used to correct for any variations in protein loading and transfer efficiency. For each protein target, the mean density of the control (PBS) sample band was assigned an arbitrary density of 1. All individual densitometry values were expressed relative to this mean.

### Effect of human sEVs T-cell activation and proliferation

The spleen was collected and immune cells were isolated according to an established protocol [43]. Immune cells were labeled with the fluorescent probe CellTraceTM Violet (CTV) (5 μmol/L, code C34557, Thermo Fisher Scientific) following the manufacturer’s instructions. CTV- Tcell were cultured in the absence or presence of Concanavalin A (ConA) (10 µg/ml) and total circulating sEVs (100 µg/ml) isolated from plasma obtained from women with normal glucose tolerance test (normal pregnancy) or gestational diabetes mellitus (GDM) in complete RPMI medium. After 4 days of culture, cells were stained with CD3-FITC (code 349201 Becton Dickinson), CD25-PE (code 341009 Becton Dickinson) and LIVE/DEAD™ Fixable Near-IR (code L10119, Thermo Fisher Scientific) and acquired on the flow cytometer Navios EX (Beckmann Coulter). The data were analyzed using the Beckman Coulter Kaluza Analysis Software. Percentage of proliferative viable CD3+ T cell was assessed by cell division based on CellTrace Violet fluorescence dilution following manufacturer’s instructions.

### Statistical analysis

Results are presented as mean ± SEM. All statistical tests were performed using Graph Pad Prism, version 9.1.1. One-way ANOVA with Bonferroni correction for multiple comparisons was used to determine significant differences. A *P*-value <0.05 was considered significant.

### Data and resource availability

The data sets generated and analyzed during the current study are available from the corresponding author upon reasonable request.

## Results

### Characterization of sEVs

**Figure 1:**
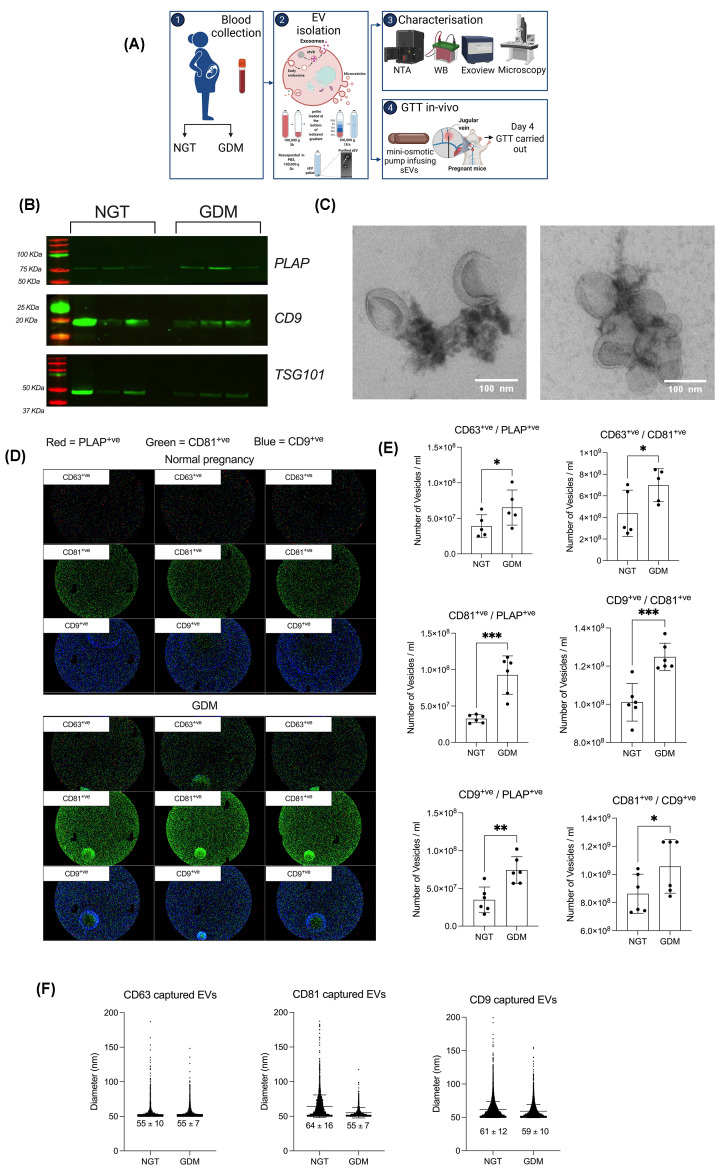
EV characterization (**A**) Schematic of the experimental design. Representative (**B**) western blot and (**C**) transmission electron microscopy images of isolated sEVs. sEVs were captured using tetraspanin specific antibodies, (**D**) analyzed with the ExoView platform and (**E**) the heterogeneity of CD63^+^, CD9^+^, CD81^+^ and PLAP^+^ sEVs between normal and GDM groups determined. (**F**) Size distribution of tetraspanin captured sEVs demonstrates no differences between normal and GDM groups. For the ExoView analysis, capture antibodies specific to EV proteins (CD63^+^, CD9^+^ and CD81^+^) immobilize EVs on the chip, and subsequently stained with three fluorescent antibodies (CD9^+^, CD81^+^ and PLAP^+^). T-test, mean ± SEM, *P<0.05, **P<0.01, ***P<0.001.

Extracellular vesicles were isolated from plasma obtained from women with normal pregnancy and GDM at the time of diagnosis (24–28 weeks of gestation) [40] (Figure 1A). EVs were characterised according to the recommendation of the International Society of Extracellular Vesicles (ISEV) by size, abundance of proteins associated with EVs, and morphology using nanoparticle tracking analysis, Western blots, and electron microscopy (Figure 1A–C). EVs were positive for CD9, TSG101 and PLAP confirming the presence of vesicles from placental origin in our preparations (Figure 1B). A preparation of vesicles of approximately 100 nm were identified, consistent with the characteristics of small EVs (Figure 1C). To investigate the heterogeneity of circulating sEVs during normal and GDM pregnancies, we performed single vesicle analysis using ExoView R100 (Nanoview Biosciences) (Supplementary Figure S1). sEVs were captured using tetraspanin specific antibodies. Analysis using ExoView shows the presence of CD63, CD9 and CD81 on captured vesicles (Figure 1D). We then assessed the heterogeneity of placenta-derived sEVs by detection of sEVs using fluorescence-tagged tetraspanin and PLAP antibodies. The CD63+ve/PLAP+ve, CD63+ve/CD81+ve, CD81+ve/PLAP+ve, CD9/CD81+ve, CD9+ve/PLAP+ve and CD81+ve/CD9+ve were significantly higher in GDM compared with normal pregnancy (*P<0.05, **P<0.005 and ***P<0.0001) (Figure 1D,E). Thus, these data suggest that GDM is associated with high circulating levels of a heterogeneous population of placental EVs, including diverse abundance of tetraspanin proteins. While the cause of this difference is not clear, these results support the robustness of detection for placenta-specific circulating sEVs based on assessment of the heterogeneity at the single EV level. Analysis of the size distribution showed an enrichment of small EV CD63+ve, CD81+ve and CD9+ve using ExoView R100; no differences were identified between NGT and GDM groups (Figure 1F). This data established that mainly small EVs are captured with tetraspanin proteins, CD9, CD81 and CD63, and no differences in the size of the EVs captured from normal compared with GDM were observed.

### Glucose tolerance and fasting plasma hormones in pregnant mice after 4-day infusion of sEVs

**Figure 2:**
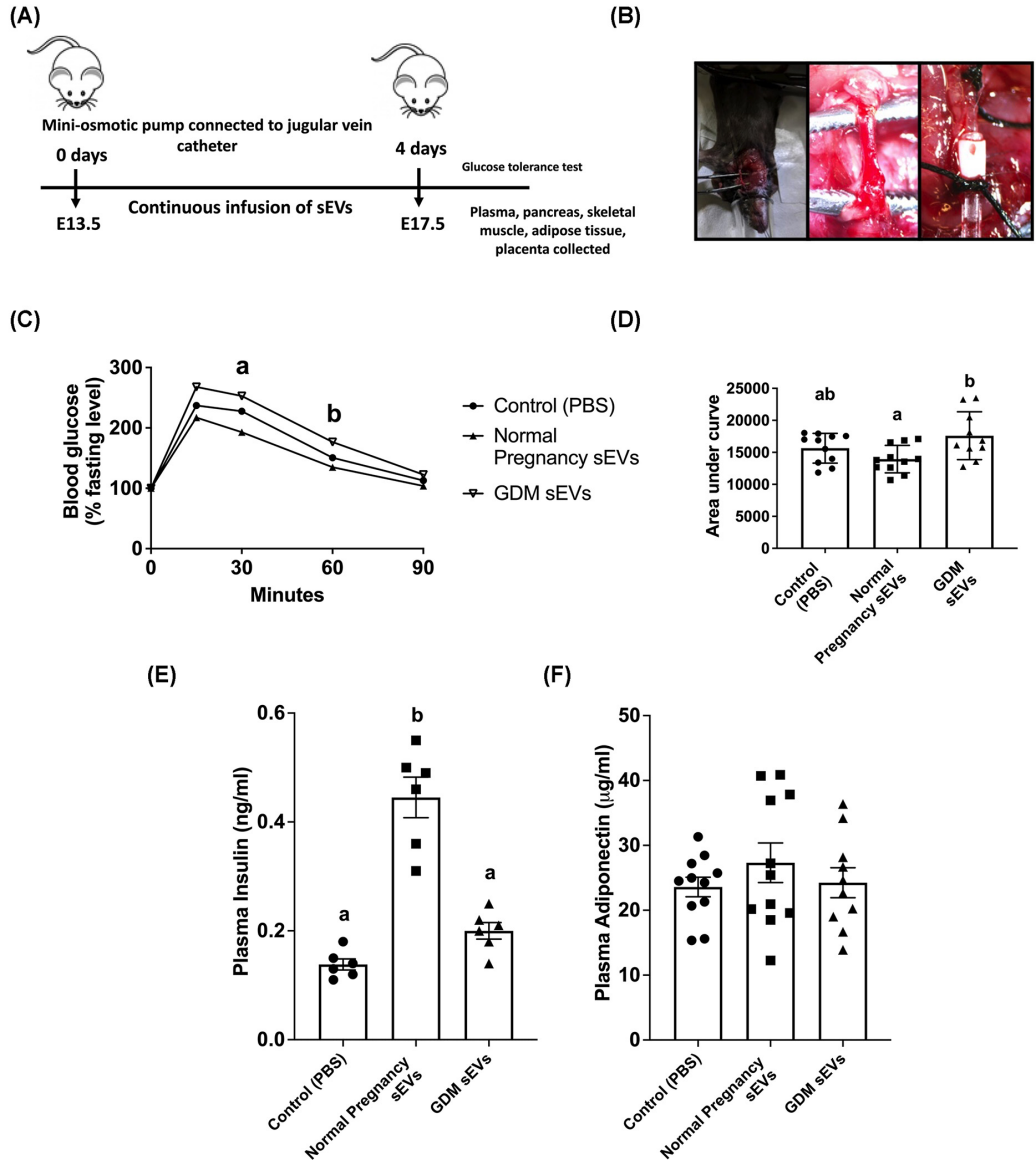
Effects of human sEV infusion on glucose homeostasis in pregnant mice (**A**) Experimental design of the *in vivo* mouse model and (**B**) surgery showing the catheter inserted into the jugular vein. Pregnant mice received a continuous 4-day infusion of PBS (control, *n*=11), or sEVs from women with normal pregnancies (*n*=11) or women with GDM (*n*=10). (**C**) Blood glucose levels were measured and (**D**) the area under the blood glucose response curve was significantly increased in mice infused with sEVs from GDM women compared with sEVs from normal pregnancies (*P*=0.02). Fasting concentration of (**E**) insulin (*n*=6/group) and (**F**) total adiponectin (*n*=10–11/group) was measured in plasma collected on day 4 of infusion. One-way ANOVA, mean ± SEM. Groups with different superscript letters are significantly different.

To determine the effect of sEV on glucose homeostasis in pregnant mice, sEVs were infused using a novel technique of chronic intravenous infusion into mice to generate a steady state circulating concentration over 4 days (Figure 2A,B). Previous studies demonstrated a steady-state concentration of human sEVs in mouse circulation via intravenous infusion [40]. Fasting blood glucose concentrations were not significantly different (*P*=0.79) between the groups (Control = 130.3 ± 3.3 mM (*n*=9), normal pregnancy sEVs = 130.5 ± 1.2 mM (*n*=11), GDM sEVs = 128 ± 3.09 mM (*n*=11), Supplementary Table S1). There was a significant difference in blood glucose levels at 30 min (*P*=0.046) and 45 min (*P*=0.026) post glucose challenge in mice receiving infusion of sEVs from women with GDM compared with healthy pregnancies (Figure 2C; *n*=10–11/group)). The area under the blood glucose curve was significantly increased in mice receiving a 4-day infusion of sEVs from women with GDM compared with mice receiving sEVs from normal pregnancies (*P*=0.02, *n*=10–11/group, Figure 2D).

After 4 days of infusion plasma was collected and hormone levels measured by ELISA. The fasting concentration of plasma insulin was significantly increased in mice receiving infusion of sEVs from women with healthy pregnancy compared with control (PBS) mice (*P*<0.0001) and mice receiving sEVs from women with GDM (*P*<0.0001) (*n*=6/group, Figure 2E). There was no significant difference between mice receiving sEVs from women with GDM compared with control (PBS) mice (*P*=0.24). There were no significant differences in the fasting concentration of total adiponectin or high molecular weight adiponectin (data not shown) in the circulation of mice after 4-day infusion of either PBS, sEVs from healthy pregnancy, or GDM (*n*=10–11/group; Figure 2F).

### Islet insulin secretion in pregnant mice after 4-day infusion of sEVs

**Figure 3:**
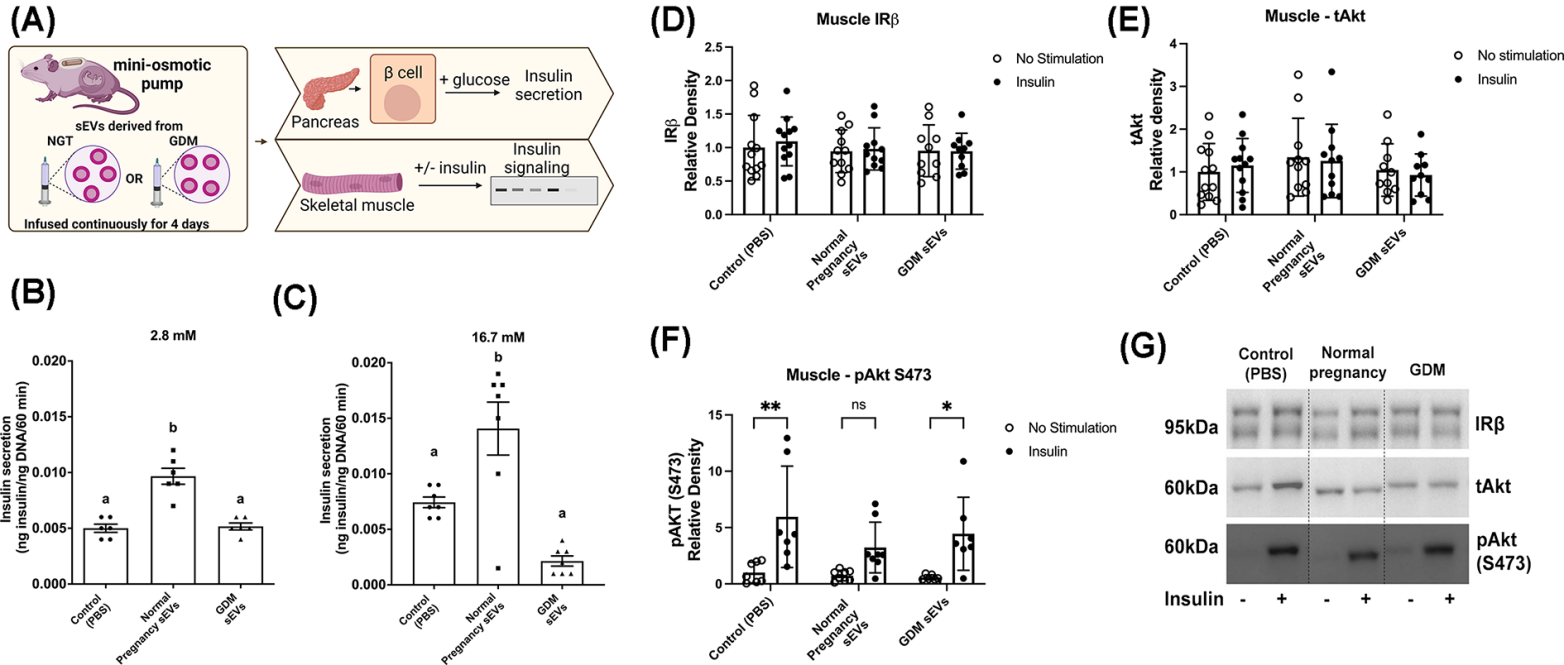
Effects of continuous infusion of human sEVs on insulin secretion and signaling in pregnant mice (**A**) Schematic of experimental design. Pregnant mice received a continuous infusion of PBS (control) or sEVs from women with normal pregnancy or GDM. Pancreatic islet cells were isolated, stimulated with (**B**) 2.8 mM (*n*=6/group) or (**C**) 16.7 mM (*n*=7/group) glucose and insulin secretion determined. The gastrocnemius muscle was collected and incubated without (no stimulation) or with insulin for 5 min and protein expression of (**D**) IRβ, (**E**) Akt and (**F**) pAkt (S473) were determined by Western blot (*n*=6–12/group). (**G**) Representative Western blots. One-way ANOVA, mean ± SEM. Groups with different superscript letters are significantly different or *P<0.05, **P<0.01.

Glucose-stimulated insulin secretion (GSIS) from islets isolated from mice infused with sEVs for 4 days was determined (*n*=6–7/group) (Figure 3A). Insulin secretion in response to 2.8 mM of glucose was increased in islets isolated from mice infused with sEVs from women with healthy pregnancies compared with mice infused with PBS (*P*<0.0001) and sEVs from women with GDM (*P*<0.0001) (Figure 3B). Similarly, there was a significant increase in insulin secretion from islets stimulated with 16.7 mM glucose in mice receiving sEVs from healthy pregnant women compared with the control (*P*=0.012) and women with GDM (*P*<0.0001) (Figure 3C). The rate of insulin secretion from islets from mice receiving GDM sEVs was not significantly different from islets isolated from animals receiving infusion of PBS when stimulated with 2.8 or 16.7 mM of glucose. This suggests that GSIS was attenuated in islets isolated from mice receiving sEVs from women with GDM.

### Insulin signaling in skeletal muscle and adipose tissue

Following 4 days of continuous infusion of PBS (control) or sEVs, mice were killed, and gastrocnemius muscle and subcutaneous adipose tissue were isolated (Figure 3A). Insulin signaling in the adipose tissue was determined by protein expression of key insulin signaling targets. There were no significant differences in expression of IRβ, tAkt, pAkt (T308) or pAkt (s473) in adipose tissue between the three groups (*n*=10/group; Supplementary Figure S2).

Gastrocnemius muscle strips were incubated with or without insulin for 5 min and protein expression of IRβ and tAkt and phosphorylation of Akt (S473) were determined. There were no significant differences between groups in basal or insulin-stimulated total expression of the insulin receptor isoform IRβ (*n*=10–12/group, Figure 3D) or expression of total Akt (*n*=10–12/group, Figure 3E). Insulin significantly stimulated the phosphorylation of Akt (S473) in control (PBS) mice (*P*=0.002) and in mice receiving infusion of sEVs from women with GDM (*P*=0.02, *n*=6–8/group, Figure 3F,G). Although insulin increased Akt (S473) phosphorylation by 77% in muscle of dams infused with sEVs from healthy pregnancies, this change failed to reach statistical significance (*n*=6–8/group, *P*=0.15, Figure 3F,G).

### Effect of sEV on immune response

**Figure 4:**
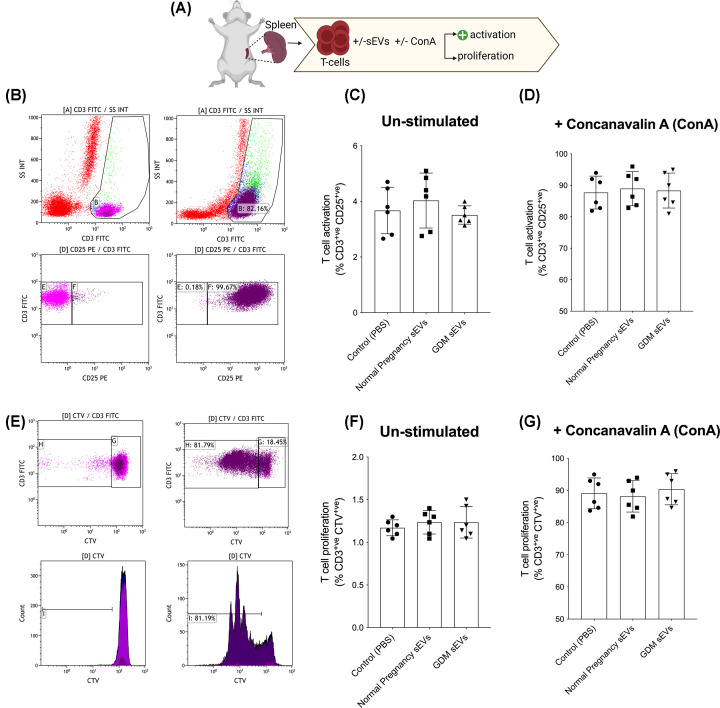
Effects of sEVs on immune response (**A**) Schematic of immunoregularity *in vivo* effect of sEVs isolated from normal and GDM pregnancies in mice. Mouse splenocytes were isolated and incubated with sEVs from normal and GDM pregnancies. (**B**) T-cell activation gating and evaluation of (**C**) unsimulated cells and (**D**) in the presence of concavalin (ConA). (**E**) T-cell proliferation gating and evaluation of (**F**) un-simulated cells and (**G**) in the presence of concavalin (ConA), one-way ANOVA, mean ± SEM.

To determine if infusion of human sEVs in pregnant mice elicits an immune response, the effect of sEVs on mouse splenocytes was determined (Figure 4A). Specifically, mouse splenocytes were isolated and incubated with sEVs from normal and GDM pregnancies, and T-cell activation and proliferation in the absence or presence of concavalin A (ConA) was assessed. No significant difference (*P*>0.05) across the groups (i.e., control (without sEVs) and treatments (+ sEVs from normal and GDM)) were observed (Figure 4B–G).

### Placental and fetal characteristics in sEV infused pregnant mice

After 4 days of sEV infusion, mice were killed and placental and fetal weights recorded. There were no significant differences in fetal, placental, fetal/placental weight ratio or litter size between the three experimental groups (Table 1). Using Western blots, the activity of placental insulin and mTOR signaling was determined. There were no significant differences in the expression of the insulin receptor β (IRβ) isoform or downstream insulin signaling targets, total Akt, pAkt (T308) or (S473) between mice receiving infusion of PBS or sEVs from healthy pregnancy or GDM (*n*=10/group, Supplementary Figure S3). There were no significant differences observed in the experimental groups in total expression or phosphorylation of the mTOR targets pS6rp (S235/236) or 4EBP1 (S65) (*n*=9–11/group, Supplementary Figure S4).

**Table 1:** Placental and fetal characteristics in mice infused with PBS (control) or sEVs from women with healthy pregnancies or GDM

	Control (PBS), mean ± SEM	Normal pregnancy sEVs, mean ± SEM	GDM sEVs, mean ± SEM	One-way ANOVA
*n*	11	11	10	
Fetal weight (g)	0.87 ± 0.03	0.83 ± 0.04	0.88 ± 0.04	0.561
Placental weight (g)	0.086 ± 0.01	0.105 ± 0.01	0.108 ± 0.03	0.245
Fetal:placental weight	11.9 ± 1.74	8.76 ± 1.01	8.58 ± 0.70	0.130
Litter size	7.91 ± 0.64	7.0 ± 0.63	7.3 ± 1.01	0.689

## Discussion

We developed a novel approach, involving continuous intravenous infusion of sEVs with a miniosmotic pump and report that, using the same concentration of sEVs, chronic infusion of sEVs isolated from GDM women causes glucose intolerance in pregnant mice. Infusion of sEVs isolated from women with healthy pregnancies into normal pregnant mice increased GSIS from pancreatic islet cells resulting in elevated levels of fasting circulating insulin. In contrast, sEVs isolated from women diagnosed with GDM failed to promote islet GSIS and did not increase fasting levels of insulin in pregnant mice. These results suggest that circulating sEVs regulate maternal glucose homeostasis in pregnancy and may contribute to the attenuated islet insulin secretion and more pronounced glucose intolerance in GDM as compared with healthy pregnancy.

Normal healthy human pregnancy is characterized by essential maternal metabolic adaptations, including increased pancreatic islet insulin secretion, elevated circulating insulin levels and insulin resistance in skeletal muscle and adipose tissue. These adaptations are believed to promote release and transport of critical nutrients, such as glucose and free fatty acids, from the maternal circulation to the fetus allowing for optimal fetal growth and development. In pregnancies complicated by GDM, maternal insulin resistance is increased further, and β-cell compensation is inadequate, resulting in various degrees of fasting hyperglycemia [3].

The half-life of circulating EVs when injected intravenously is short, due to rapid secretion/clearance [44]. Consequently, we established a novel mouse model in which sEVs were administered by continuous infusion over 4 days via mini-osmotic pumps leading to a steady concentration of human sEVs in the mouse circulation. In our previous publication [40], where we used the same approach to chronically infuse sEVs by osmotic pump and an indwelling jugular vein catheter, we confirmed successful delivery of EVs into the circulation of the recipient mouse. First, we showed that the protein expression of the EV marker CD63 in plasma was markedly increased following administration of human sEVs when compared with PBS infused control animals. We also provided indirect support for delivery of EV cargo to target tissues in mice infused with sEVs as we demonstrated expression of miRNAs from the C19 cluster, only expressed in primates, in the skeletal muscle of mice infused with human sEVs. An additional strength of the present study is that we used sEVs from women which were isolated at the time of GDM diagnosis, between 24 and 28 weeks gestation, avoiding possible confounding effects due to different GDM treatments.

In the present study, sEVs isolated from healthy pregnant women increased glucose stimulated islet insulin secretion and elevated fasting plasma insulin in pregnant mice, in agreement with our previous studies in nonpregnant mice [40]. However, in contrast with nonpregnant mice [40], chronic infusion of sEVs isolated from normal pregnant women did not cause a more pronounced glucose tolerance or muscle insulin resistance as compared with pregnant mice infused with PBS. This distinct difference in the metabolic effects of sEVs isolated from normal pregnant women between nonpregnant and pregnant mice is likely due to the basal muscle insulin resistance and glucose intolerance already present in pregnant animals. In addition to the human sEVs infused into their circulation, pregnant mice at E13.5-17.5 also have endogenous sEVs, including from maternal and placental sources, and endocrine signals that are associated with pregnancy, including placental hormones, that contribute to maternal insulin resistance. Nonpregnant mice do not have the changes associated with pregnancy and therefore demonstrated a more robust response to sEVs from pregnant women.

Pregnant mice that received sEVs from GDM women were more glucose intolerant than pregnant mice infused with sEVs from normal healthy pregnant women. This is likely due to the lack of stimulatory effect of sEVs from GDM women on islet insulin secretion, which is reflected by the lower fasting insulin in these animals as compared with pregnant mice infused with sEVs from normal pregnant women. These observations mirror the effects of administration of sEVs isolated from GDM women in nonpregnant mice that we reported previously [40]. Our results are consistent with the possibility that the β-cell dysfunction and more pronounced glucose intolerance in GDM women, as compared with normal pregnant women, is mediated, at least in part, by sEVs. In contrast with nonpregnant mice [40], chronic infusion of sEVs isolated from GDM women did not cause a more pronounced muscle insulin resistance as compared with pregnant mice infused with PBS. We speculate that these differences in responses are due to the high basal insulin resistance in pregnant as compared to non-pregnant animals.

Adipose tissue, predominantly made up of adipocytes, is an energy store and an endocrine organ that releases adipokines that have a role in glucose and lipid metabolism. During pregnancy, there is an accumulation of adipose tissue to support nutrient delivery to the fetus. However, obesity, which is associated with an expansion of adipose tissue and is a major risk factor for GDM, is linked with inflammation and insulin resistance [45,46]. Adiponectin, an adipokine released by adipocytes, promotes insulin sensitivity, and has anti-inflammatory and antiatherogenic effects. Reduced levels of adiponectin are associated with obesity, insulin resistance and GDM [47,48]. We did not observe changes in adipose tissue insulin sensitivity or in fasting concentrations of circulating adiponectin in mice infused with sEVs isolated from normal term or GDM women. Unlike skeletal muscle, we did not stimulate adipose tissue with insulin and therefore, do not know if sEV infusion in mice affected insulin stimulated adipose tissue insulin signaling. As we did not measure adipose tissue insulin sensitivity in our previous study [40], the effect of sEVs from normal pregnant or GDM women on adipose tissue signaling in non-pregnant mice is unknown. However, it is possible that the lack of response in adipose tissue signaling in the current study reflects that maternal plasma sEVs do not target adipose tissue. Alternatively, adipose tissue insulin resistance caused by pregnancy may have limited our ability to demonstrate any effect of administered sEVs on adipose insulin signaling in the current study. Future studies are required to determine the effect of sEVs in pregnancy on adipose tissue insulin resistance.

One limitation with the present study are the differences observed between murine and human pregnancies, which include multifetal pregnancies in mice, differences in endocrine regulation and miRNA expression [49]. Although the C19 miRNA cluster is primate specific and there are differences in the C14MC between humans and mice, our study demonstrates that human sEVs infused into mice have an effect on maternal glucose tolerance and GSIS, suggesting that these miRNAs might not be essential for these physiological effects [50]. Administration of human sEVs to mice does not elicit toxic or inflammatory responses in these animals [51]. Our study demonstrated that administration of human sEVs from normal pregnancy and pregnancies complicated by GDM do not stimulate an immunomodulatory effect *in vitro*, determined by lack of activation and proliferation of T cells isolated from mouse splenocytes. These findings are in agreement with the literature, suggesting that administration of human EVs to experimental animals is well tolerated and does not cause toxic effects or elicit immune responses [51,52]. Because we did not determine the effect of sEVs on immune response *in vivo* we cannot completely exclude an immune response in response to the infused human sEVs in our mice. However, our *in vitro* data and the literature suggest that this is unlikely.

There were no differences in litter size, number of absorptions or placental or fetal growth in pregnant mice infused with sEVs, as compared with PBS infused mice. This suggests that administration of human sEVs has no general deleterious effect on pregnancy in mice. Consistent with the lack of fetal growth phenotype, placental insulin and mTOR signaling was unaffected by administration of sEVs from healthy pregnant or GDM women. Although GDM in women increases the risk of fetal overgrowth, this was not replicated in pregnant mice infused with sEVs from GDM women. The glucose intolerance caused by sEV infusion in our pregnant mice was likely too mild to promote accelerated fetal growth. Moreover, although GDM women typically have elevated insulin as compared to normal pregnant women, which may stimulate fetal growth, infusion of sEVs isolated from GDM women in our pregnant mice did not replicate hyperinsulinemia. Several studies have explored the effect of EVs *in vivo* using different routes of administration, type of buffer, concentration of EVs, injection regiment, etc [53–55]. The most common route of administration is via a tail-vein injection (intravenous), with EVs diluted in sterile PBS ranging in volumes of 80–200 µl containing 5–400 µg of total EV proteins (Supplementary Table S2). Progress in the field is hindered by a lack of standardized EV concentration used in *in vivo* experiments, which need to be considered to analyse and interpret the data. In the present study, we used a novel approach, involving continuous venous infusions of sEVs with a miniosmotic pump which mimics the continuous secretion of EVs *in vivo*. The number of circulating sEVs is increased in pregnancy and further increased in women with GDM; however, in the present study we infused the same concentration of sEVs from each group into the circulation of the mice. This suggests that the results observed are not solely due to an increased number of circulating sEVs but most likely due to the differing content of the sEVs between groups. The placenta releases sEVs into the maternal circulation across gestation and it is known that the population of circulating sEVs is made up of ∼20% placental sEVs at term [23,56]. The source of the remaining percentage of circulating sEVs is unknown, although it is probable that these are of maternal origin, including from adipose tissue [57]. However, further in-depth investigation is required to delineate the origin of the sEVs circulating in maternal plasma during pregnancy. The placenta and the sEVs released from it contain miRNAs, including the C14 (C14MC) and C19 miRNA clusters (C19MC) [58] and placental sEVs from pregnancies complicated with GDM have a specific set of miRNAs which are associated with skeletal muscle insulin signaling [38,39]. Placental sEVs are also enriched in proteins, such as PAPP-A and CAMK2β, and in GDM these proteins are associated with inflammation and metabolic pathways [59]. We speculate that the effects on sEVs isolated from normal pregnant and GDM women in the present study may be mediated by placental sEVs, however further studies are required to test this hypothesis.

In conclusion, our results suggest that circulating sEVs regulate maternal glucose homeostasis in pregnancy and may contribute to attenuated islet insulin secretion and more pronounced glucose intolerance in GDM compared with normal pregnancy.

## Clinical perspectives

- GDM is associated with a higher concentration of sEVs and linked to maternal glucose intolerance. However, the mechanism by which sEVs regulate maternal glucose homeostasis in pregnancy is largely unknown.
- Using a novel approach, involving continuous intravenous infusion of sEVs with a miniosmotic pump, we demonstrate that sEVs isolated from GDM women cause glucose intolerance in pregnant mice because of its impaired ability to stimulate compensatory insulin secretion.
- Our findings suggest that circulating sEVs contribute to the attenuated insulin secretion and more pronounced glucose intolerance which underlie the pathology of GDM.

## Supplementary Material

Supplementary Figures S1-S4 and Tables S1-S2

## Data Availability

Some or all datasets generated during and/or analyzed during the present study are not publicly available but are available from the corresponding authors on reasonable request.

## Open Access

Open access for this article was enabled by the participation of University of Queensland in an all-inclusive *Read & Publish* agreement with Portland Press and the Biochemical Society under a transformative agreement with CAUL.

## Abbreviations

BSA: bovine serum albumin
GDM: gestational diabetes mellitus
NGT: normal glucose tolerant
EV: extracellular vesicle
GSIS: glucose stimulated insulin secretion
GTT: glucose tolerance test
IRβ: insulin receptor β
IRS1: insulin receptor substrate 1
mTOR: mammalian target of rapamycin
PAPP-A: pregnancy associated plasma protein A
CAMK2β: calcium/calmodulin-dependent protein kinase II beta
TSG101: tumor susceptibility gene 101
OGTT: oral glucose tolerance test
MVB: multivesicular bodies
NTA: Nanoparticle Tracking Analysis
PLAP: placental alkaline phosphatase
sEV: small extracellular vesicle
ELISA: enzyme linked immunosorbent assay
