# Supplementary material for: A novel technique using chronic infusion of small extracellular vesicles from gestational diabetes mellitus causes glucose intolerance in pregnant mice
Source: Clin Sci (Lond). 2022 Nov 3;136(21):1535–49. doi: 10.1042/CS20220484 (PMC9638966; doi:10.1042/CS20220484)
Supplement: Supplementary Figures S1-S4 and Tables S1-S2 [file CS-2022-0484_supp.pdf]

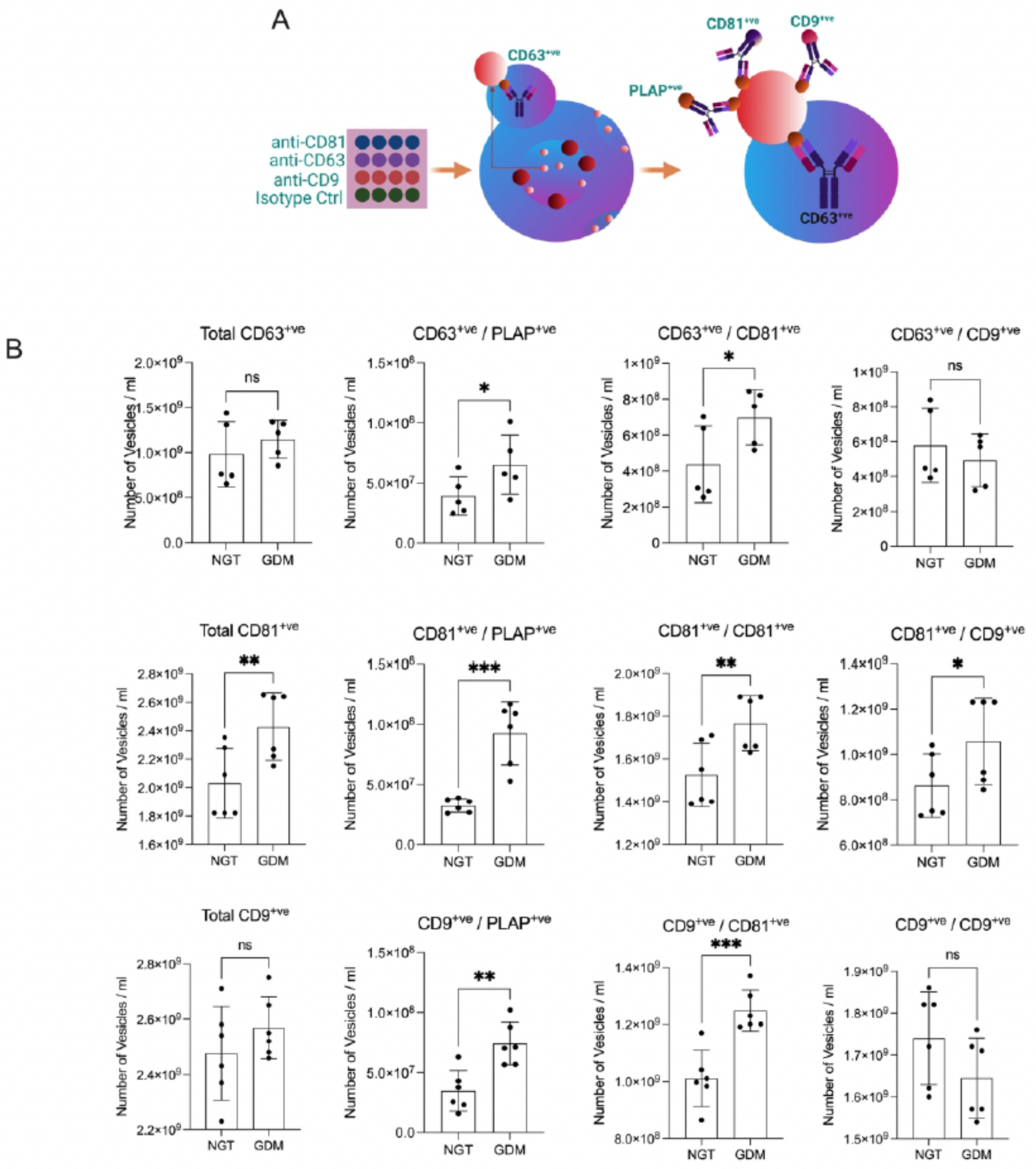

Supplemental Figure 1

Supplemental Figure 1. Single particle analysis. Particle concentrations were obtained using ExoView R100 (Nanoview Biosciences) analysis. A) schematic representation of a typical ExoView assays use generic extracellular vesicles (EV) markers to capture general EV from solution (i.e., tetraspanin: CD63. CD81, and CD9). Captured EV can then be probed for other proteins (e.g., PLAP) that may indicate origin or function. B) Vesicle count using CD63, CD81 or CD9 captured antibody on circulating EV from NGT and GDM and detected using CD9, PLAP or CD81. In B, *p < 0.05; **p < 0.01; ***p < 0.001.

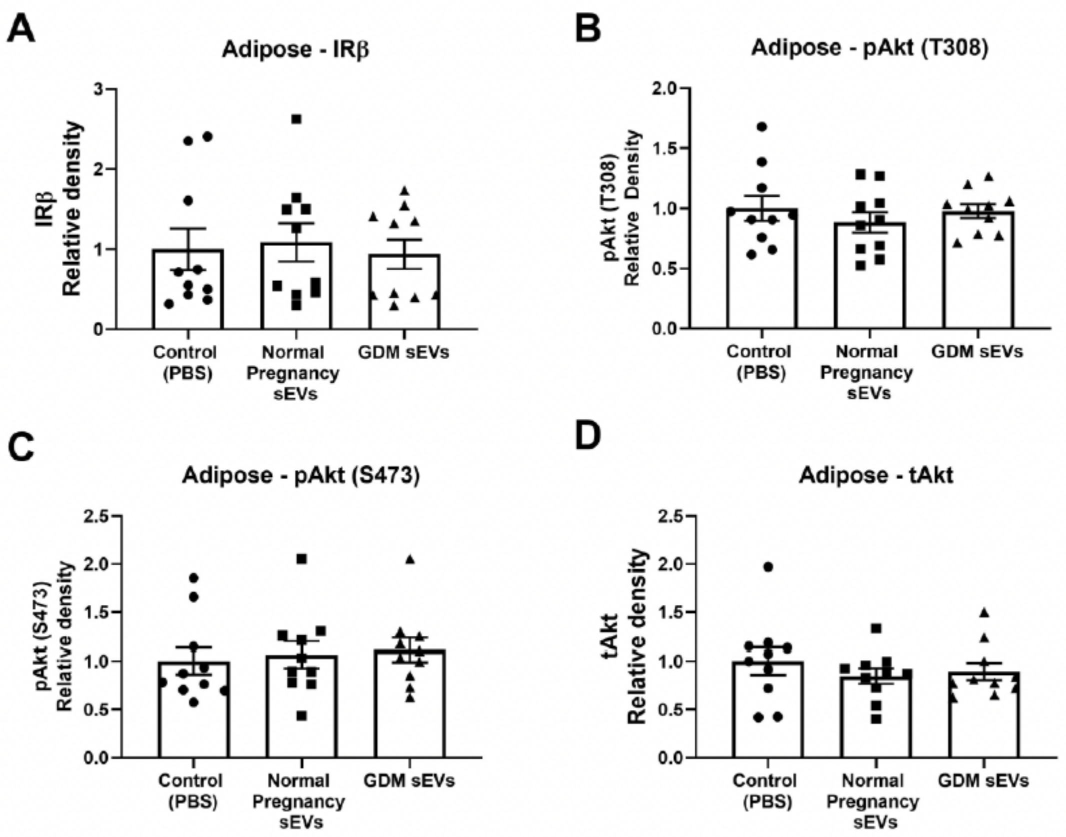

Supplemental Figure 2

Supplemental Figure 2. Insulin signaling in adipose tissue. Adipose tissue was collected after 4 days of continuous infusion of PBS or sEV from healthy women or women with GDM in pregnant mice (n=10/group). Insulin signaling targets (A) IRβ, (B) pAkt (S473), (C) pAkt (T308) and (D) tAkt were determined by western blot. Data shown as mean ± SEM, one-way ANOVA.

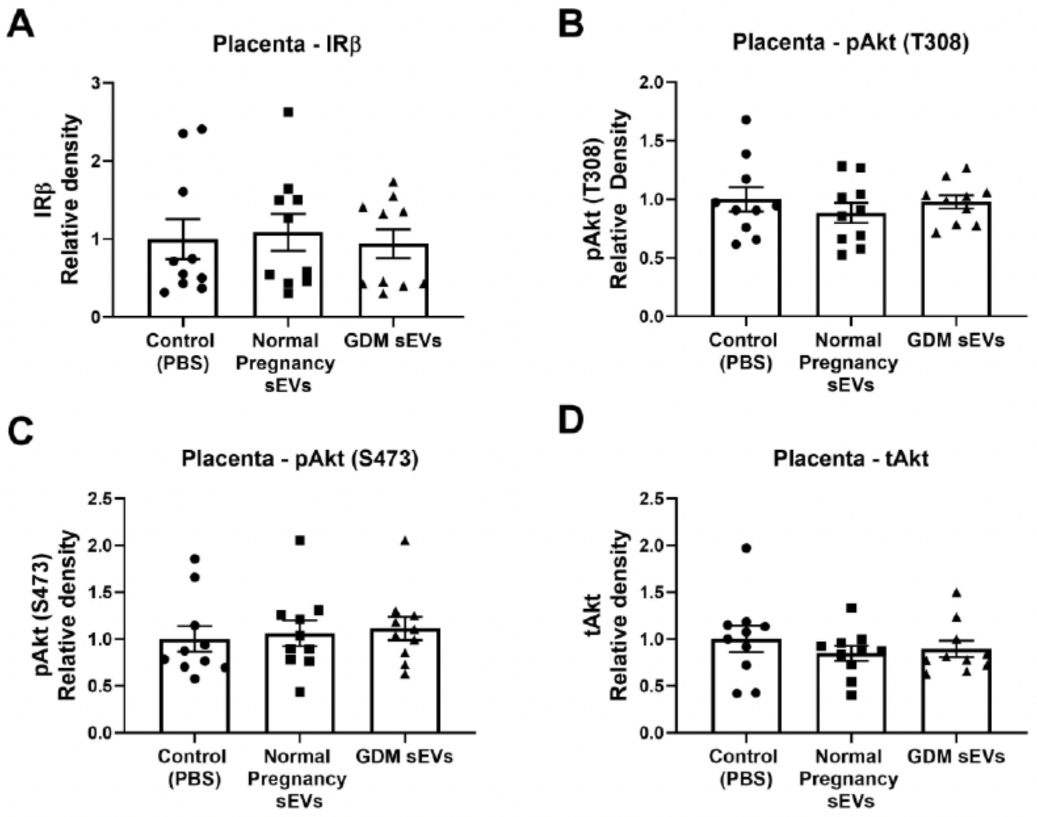

Supplemental Figure 3

Supplemental Figure 3. Insulin signaling in placental tissue. Placental tissue was collected from pregnant mice after 4 days of continuous PBS or sEV infusion from healthy women or women with GDM (n=10/group). Insulin signaling targets (A) IRβ, (B) pAkt (S473), (C) pAkt (T308) and (D) tAkt were determined by western blot. Data shown as mean ± SEM, one-way ANOVA.

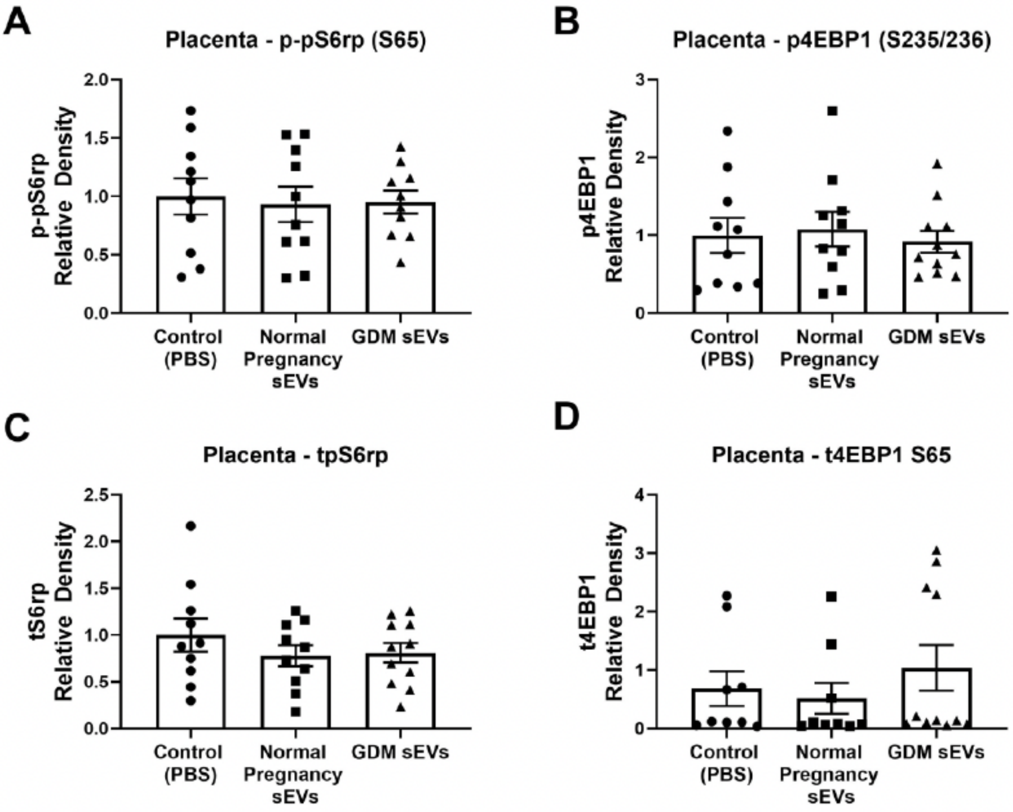

Supplemental Figure 4

Supplemental Figure 4. mTOR signaling in placental tissue. Placental tissue was collected from pregnant mice after 4 days of continuous PBS or sEV infusion from healthy women or women with GDM. mTOR signaling targets (A) total pS6rp, (B) phosphorylated pS6rp (S65), (C) total 4EBP1 and (D) phosphorylated 4EBP1 (S235/236) were determined by western blot (n=9-11/group). Data shown as mean ± SEM, one-way ANOVA.

Supplementary Table 1. Fasting blood glucose concentrations

Minutes	Control (PBS) (mM)	Healthy Pregnancy (mM)	GDM (mM)	ANOVA
**0**	130.3 ± 3.3	130.5 ± 2.2	128 ± 3.1	0.786
**15**	237.2 ± 17.8	217.1 ± 10.2	267.9 ± 17.1	0.083
**30**	227.7 ± 16.6	192.9 ± 11.1	253.0 ± 20.4	0.046*
**45**	150.7 ± 6.8	134.9 ± 7.6	176.7 ± 15.3	0.026*
**90**	112.9 ± 4.2	103.8 ± 5.0	122.7 ± 8.6	0.109

Supplemental Table 2. The most common route of administration is via a tail vein injection (intravenous). Extracellular vesicles (EV) were diluted in sterile PBS ranging in volumes of 80 – 200 µL containing 5-400 µg of total EV proteins.

Route of administration	Excipient	EV Dose	Volume	Injection regiment	Aim of project	Animal strain	EV labelling	Reference
Tail vein injection	5% glucose	150 µg	80 µL	1 dose	siRNA delivery	C57BL/6 male 8-10 week old	NA	[1]
Osmotic pumps	PBS	2.7 x 10^{12}	100µl PBS	infused continuously over 4 days	Effect on glucose homeostasis	Non-pregnant female C57/Bl6	NA	[2]
Tail vein injection	-	~5 µg	-	1 dose	*In vivo* tracking	C57BL/6, BALB/c (*in vivo* imaging)	PKH26 red fluorescent	[3]
Retro-orbital vein or tail vein injection	PBS	100 µg	-	1 dose	*In vivo* EV bioluminescence	Athymic Nude Mouse	Endogenously labelled exosome-Luciferase and biotin	[4]
Tail vein injection	PBS	1.5x10^{10} particles/gram body weight (p/g), 1.0x10^{10} p/g and 0.25x10^{10} p/g	-	1 dose	Biodistribution	Female NMRI or C57BL/6	Fluorescent lipophilic tracer DiR	[5]
Intradermal	Saline	3-5 µg of exosomes	-	1 dose	Drug delivery	DBA/2J, BALB/c		[6]
IV	-	~5 µg	-	1 dose	Macrophage dependent clearance	BALB/c 5 week old male	PKH26	[7]
Tail vein injection	5% glucose or PBS with 5% glucose?	150 µg	-	-	Methods paper	C57BL/6 mice	Cellmask	[8]
Tail vein injection	PBS	400 µg	200 µL		Safety profile	Sprague-Dawley rats		[9]

Tail vein injection	PBS	2×10^{11}	200 µL	1 dose	If skeletal muscle exosomes can be taken up by pancreatic beta cells	C57BL/6 male	Fluorescent lipophilic tracer DiR	[10]
Tail vein		10 µg		2 doses. Day 5 and day 16	Investigate the mechanism of action of exosome in vivo	Lewis rats	PKH26	[11]
Tail vein	PBS	10 µg	100	1 dose	Functionality of exosome	C57BL/6	Fluorescent lipophilic tracer DiR	[12]
Different wound sites		5 or 50 µg	200 µL	1 dose	Exosome in wound healing	Diabetic B6.Leprdb/db mice	PKH67	[13]
Tail vein	PBS	2 µg/g body weight	150 µL	1 dose	Exosomal miR-223 in cardioprotection	C57BL/6	PKH26	[14]
Subcutaneously	PBS	100 µg	200 µL	Twice a week for 50 days	Role of CML derived exosome in tumour growth	NOD/SCID, 4-5 weeks	NA	[15]
IV	PBS		150 µL	6 doses at 48 hr intervals	Serum-derived exosomes from highly metastatic breast cancer transfer the metastatic capacity	WT BALB/c	NA	[16]
Right hand paw	PBS	10 µg	50 µL	1 dose	Plasma-derived MHCII+ exosomes from tumor-bearing mice suppress tumor antigen-specific immune responses	C57BL/6	NA	[17]
Skin tissue	HBSS	10^{6}	50 µL	1 dose	Tracking of tumour extracellular vesicle delivery	C57BL/6	GFP and GlucB via lentiviral	[18]
Peri-infarct sites	Iscove's Modified	2.8×10^{9} and 1.56×10^{9}	40 µL	1 dose	Exosome in cardiac regeneration	SCID	NA	[19]

	Dulbecco's (IMDM)							
Intranasally	PBS	25 µg		1 dose	Exosome from Mycobacteria-infected mice recruit and activate immune cells	C57BL/6	NA	[20]
Tail vein	PBS	10 µg	100-200 µL PBS	3 times / week for 3 weeks	Melanoma exosomes educate bone marrow progenitor cells toward a pro-metastatic phenotype through MET	C57BL/6	NA	[21]
Intrasplenically	HBBS	10 µg		Every 3 days for 28 days	Exosome in colorectal cancer liver metastasis	Balb/c	NA	[22]
Intraperitoneal		10 µg	100 µL	1 dose	Intestinal epithelial exosomes carry MHC class II/peptides able to inform the immune system in mice	C3H/HeN	NA	[23]
Intraperitoneally	PBS	25 µg	200 µL	1 dose	DC derived exosomes induce protection against infection in mice	BALB/c	NA	[24]
Intraperitoneally		30 µg		Day 2,4,6	define the effect of granulocytic MDSC-derived exosomes (G-MDSC exo) in dextran sulphate sodium (DSS)-induced murine colitis	C57BL/6	NA	[25]

Footpad	PBS	50 µg	50 µL	1 dose	Exosomes Released by Melanoma Cells Prepare Sentinel Lymph Nodes for Tumor Metastasis	B16-F10	DIR or DIL	[26]
Intravenously		100 µg		2 doses / week for 3 weeks	Contribution of MyD88 to the tumor exosome-mediated induction of myeloid derived suppressor cells	B6 wild-type mice or MyD88 knockout	NA	[27]
Subcutaneously	PBS	0.3 µg	50 µL	1 dose / day for 4 days. Every other day for 15 days	Mesenchymal Stem Cells Secrete Immunologically Active Exosomes	C57BL/6J	NA	[28]
Subcutaneously	Saline	2.5 ± 5 µg		3 times at 2-week intervals	Immune protection effect of exosomes against attack of L1210 tumor cells	DBA/2	NA	[29]

References

1. Alvarez-Erviti, L., et al., *Delivery of siRNA to the mouse brain by systemic injection of targeted exosomes.* Nat Biotech, 2011. **29**(4): p. 341-345.
2. James-Allan, L.B., et al., *Regulation of glucose homeostasis by small extracellular vesicles in normal pregnancy and in gestational diabetes.* FASEB J, 2020. **34**(4): p. 5724-5739.
3. Takahashi, Y., et al., *Visualization and in vivo tracking of the exosomes of murine melanoma B16-BL6 cells in mice after intravenous injection.* Journal of Biotechnology, 2013. **165**(2): p. 77-84.
4. Lai, C.P., et al., *Dynamic Biodistribution of Extracellular Vesicles in Vivo Using a Multimodal Imaging Reporter.* ACS Nano, 2014. **8**(1): p. 483-494.
5. Wiklander, O.P.B., et al., *Extracellular vesicle in vivo biodistribution is determined by cell source, route of administration and targeting.* Journal of Extracellular Vesicles, 2015.

6. Zitvogel, L., et al., *Eradication of established murine tumors using a novel cell-free vaccine: dendritic cell derived exosomes.* Nat Med, 1998. **4**(5): p. 594-600.
7. Imai, T., et al., *Macrophage-dependent clearance of systemically administered B16BL6-derived exosomes from the blood circulation in mice.* Journal of Extracellular Vesicles, 2015. **4**: p. 10.3402/jev.v4.26238.
8. El-Andaloussi, S., et al., *Exosome-mediated delivery of siRNA in vitro and in vivo.* Nat. Protocols, 2012. **7**(12): p. 2112-2126.
9. Sun, L., et al., *Safety evaluation of exosomes derived from human umbilical cord mesenchymal stromal cell.* Cytotherapy, 2016. **18**(3): p. 413-422.
10. Jalabert, A., et al., *Exosome-like vesicles released from lipid-induced insulin-resistant muscles modulate gene expression and proliferation of beta recipient cells in mice.* Diabetologia, 2016. **59**(5): p. 1049-1058.
11. Li, X.-L., et al., *Exosomes derived from atorvastatin-modified bone marrow dendritic cells ameliorate experimental autoimmune myasthenia gravis by up-regulated levels of IDO/Treg and partly dependent on FasL/Fas pathway.* Journal of Neuroinflammation, 2016. **13**(1): p. 8.
12. Liu, H., et al., *Exosomes derived from dendritic cells improve cardiac function via activation of CD4+ T lymphocytes after myocardial infarction.* Journal of Molecular and Cellular Cardiology, 2016. **91**: p. 123-133.
13. Geiger, A., A. Walker, and E. Nissen, *Human fibrocyte-derived exosomes accelerate wound healing in genetically diabetic mice.* Biochemical and Biophysical Research Communications, 2015. **467**(2): p. 303-309.
14. Wang, X., et al., *Exosomal miR-223 Contributes to Mesenchymal Stem Cell-Elicited Cardioprotection in Polymicrobial Sepsis.* Scientific Reports, 2015. **5**: p. 13721.
15. Raimondo, S., et al., *Chronic myeloid leukemia-derived exosomes promote tumor growth through an autocrine mechanism.* Cell Communication and Signaling, 2015. **13**(1): p. 8.
16. Gorczynski, R.M., N. Erin, and F. Zhu, *Serum-derived exosomes from mice with highly metastatic breast cancer transfer increased metastatic capacity to a poorly metastatic tumor.* Cancer Medicine, 2016. **5**(2): p. 325-336.
17. Yang, C., et al., *Plasma-derived MHCII+ exosomes from tumor-bearing mice suppress tumor antigen-specific immune responses.* European journal of immunology, 2012. **42**(7): p. 1778-1784.
18. Lai, C.P., et al., *Visualization and tracking of tumour extracellular vesicle delivery and RNA translation using multiplexed reporters.* Nature Communications, 2015. **6**: p. 7029.
19. Ibrahim, Ahmed G.-E., K. Cheng, and E. Marbán, *Exosomes as Critical Agents of Cardiac Regeneration Triggered by Cell Therapy.* Stem Cell Reports, 2014. **2**(5): p. 606-619.
20. Singh, P.P., et al., *Exosomes Isolated from Mycobacteria-Infected Mice or Cultured Macrophages Can Recruit and Activate Immune Cells In Vitro and In Vivo.* The Journal of Immunology, 2012.
21. Peinado, H., et al., *Melanoma exosomes educate bone marrow progenitor cells toward a pro-metastatic phenotype through MET.* Nat Med, 2012. **18**(6): p. 883-891.
22. Wang, X., et al., *Investigation of the roles of exosomes in colorectal cancer liver metastasis.* Oncology reports, 2015. **33**(5): p. 2445-2453.
23. Van Niel, G., et al., *Intestinal epithelial exosomes carry MHC class II/peptides able to inform the immune system in mice.* Gut, 2003. **52**(12): p. 1690-1697.

24. Colino, J. and C.M. Snapper, *Dendritic Cell-Derived Exosomes Express a Streptococcus pneumoniae Capsular Polysaccharide Type 14 Cross-Reactive Antigen That Induces Protective Immunoglobulin Responses against Pneumococcal Infection in Mice.* Infection and Immunity, 2007. **75**(1): p. 220-230.
25. Wang, Y., et al., *Exosomes released by granulocytic myeloid-derived suppressor cells attenuate DSS-induced colitis in mice.* Oncotarget, 2016. **7**(13): p. 15356-15368.
26. Hood, J.L., R.S. San, and S.A. Wickline, *Exosomes released by melanoma cells prepare sentinel lymph nodes for tumor metastasis.* Cancer research, 2011. **71**(11): p. 3792-3801.
27. Liu, Y., et al., *Contribution of MyD88 to the tumor exosome-mediated induction of myeloid derived suppressor cells.* The American journal of pathology, 2010. **176**(5): p. 2490-2499.
28. Zhang, B., et al., *Mesenchymal stem cells secrete immunologically active exosomes.* Stem cells and development, 2013. **23**(11): p. 1233-1244.
29. Bu, N., et al., *Immune protection effect of exosomes against attack of L1210 tumor cells.* Leukemia & lymphoma, 2006. **47**(5): p. 913-918.
